# Fibre digestibility, abundance of faecal bacteria and plasma acetate concentrations in overweight adult mares

**DOI:** 10.1017/jns.2014.8

**Published:** 2014-05-07

**Authors:** Megan L. Shepherd, Monica A. Ponder, Amy O. Burk, Stewart C. Milton, William S. Swecker

**Affiliations:** 1 Department of Large Animal Clinical Sciences, Virginia-Maryland Regional College of Veterinary Medicine, Virginia Tech, Duck Pond Drive (0442), Blacksburg, VA 24061, USA; 2 Department of Food Science and Technology, Virginia Tech, Blacksburg, VA 24061, USA; 3 Department of Animal and Avian Sciences, University of Maryland, College Park, MD 20742, USA

**Keywords:** Body condition score, Digestible energy, Faecal bacteria, Horses, ADF, acid-detergent fibre, BCS, body condition score, DE, digestible energy, DMI, DM intake, NDF, neutral-detergent fibre, OG, orchard grass, rDNA, ribosomal DNA, rRNA, ribosomal RNA, VFA, volatile fatty acid

## Abstract

The purpose of the present study was to compare digestibility of grass hay, faecal and plasma volatile fatty acid (VFA) concentrations, and faecal bacterial abundance in overweight and moderate-condition mares. Five overweight adult mixed-breed mares and five adult mixed-breed mares in moderate condition were housed individually and limit-fed orchard grass (*Dactylis glomerata*) hay at 20 g/kg body weight (as fed) daily for 14 d. Forage DM and fibre digestibility were determined using AOAC methods; digestible energy was measured using bomb calorimetry; plasma and faecal VFA concentrations were determined by use of GC and MS; faecal Firmicutes, Bacteroidetes, *Fibrobacter succinogenes*, *Ruminococcus flavefaciens* and total bacteria abundance was determined by quantitative real-time PCR using previously designed phylum-specific 16S ribosomal RNA gene primers. No differences in hay digestibility, faecal VFA concentrations or faecal bacterial abundance were detected between overweight and moderate-condition mares. Mean plasma acetate concentrations were higher (*P* = 0·03) in overweight (1·55 (range 1·43–1·65) mmol/l) *v.* moderate-condition (1·39 (range 1·22–1·47) mmol/l) mares. We conclude that the higher plasma acetate in overweight mares should be further investigated as a potential link between gut microbes and obesity in horses.

A total of 51 % of adult horses in southwest Virginia state, USA, are overweight or obese^(^^1^^)^ and with similar findings in Scotland^(^^2^^)^ and the UK^(^^3^^)^, the rate of obesity may be similar in other equine populations. Obesity is a critical problem for the horse population due to the many negative downstream effects on health, welfare and performance. Negative health effects of obesity in horses include reduced reproductive performance^(^^4^^,^^5^^)^, reduced evaporative cooling and reduced athletic performance^(^^6^^)^, and insulin resistance^(^^7^^–^^9^^)^. The latter increases the risk of laminitis, a painful and debilitating condition of the equine hoof^(^^10^^,^^11^^)^.

Many factors influence the development of an overweight and obese state, but, simply, weight gain occurs when a surplus of energy is consumed relative to energy utilisation. Interestingly, Thatcher *et al.*^(^^1^^)^ reported that the obese horses in the southwest Virginia study were consuming a forage-based diet, suggesting that horses are becoming overweight and obese on forage alone and not necessarily highly digestible commercial feeds and grains. Forage is high in fibre, which is not digested by mammalian enzymes due to the β-glycosidic bonds linking monosaccharide residues; however, fibres are digested by micro-organisms in the gastrointestinal tract (also known as gut microbes or gut microbiota), specifically the caecum and colon, of the horse^(^^12^^)^. These gut microbes produce usable products (i.e. volatile fatty acids; VFA) from otherwise indigestible substrates. Acetate is the dominant VFA produced by equine gut microbes^(^^13^^–^^17^^)^. Diet and diet change influence faecal VFA concentrations in horses in that the acetate:propionate ratio is generally lower with increasing grain starch in the diet^(^^17^^,^^18^^)^. Furthermore, Julliand *et al.*^(^^19^^)^ reported that the concentration of fibrolytic bacteria and acetate production were lower in the caecum and colon of horses fed hay plus barley *v.* horses fed hay alone.

Obese humans and rodents appear to have a unique gut microbiota as compared with their lean counterparts^(^^20^^,^^21^^)^. Conventionally raised mice (those with gut microbes) have greater diet-induced weight gain than their germ-free (those without gut microbes) mouse counterparts^(^^22^^,^^23^^)^. Furthermore, Turnbaugh *et al.*^(^^24^^)^ reported that obese mice (*ob/ob*) have higher caecal acetate concentrations than non-obese wild-type mice. The role of VFA such as acetate on fat mass may be two-fold: VFA serve as an energy source and as ligands for G protein-coupled receptors with subsequent inhibition of lipolysis and stimulation of adipogenesis^(^^25^^,^^26^^)^. In horses, plasma acetate may be aerobically oxidised and directly used for energy^(^^27^^)^ or stored as TAG in adipose and skeletal tissue^(^^28^^)^.

The equine gut microbiota has received increasing attention due to the importance of gut microbes in equine health. The Firmicutes phylum dominates the hindgut (caecum and large colon) and faecal microbiome in horses (44–72 % of total bacteria)^(^^29^^,^^30^^)^. The abundance of Bacteroidetes in horses varies between 4 and 49 % of total bacteria^(^^31^^–^^33^^)^. In obese mice, pigs and human subjects there is an association between increased relative abundance of the Firmicutes phylum, along with a reduction in the relative abundance of the Bacteroidetes phylum^(^^20^^,^^21^,^34^^–^^36^^)^. While this is an area of controversy due to the inconsistency in abundance of these two phyla in obese *v.* lean individuals, there is also variation between studies with respect to host species, samples evaluated (i.e. faecal *v.* intestine lumen *v.* intestinal mucosa), region of the gastrointestinal tract evaluated, and time point relative to obesity^(^^37^^,^^38^^)^. Nevertheless, these phyla continue to be associated with obesity in recent studies^(^^39^^,^^40^^)^ and have not yet been evaluated relative to obesity in the horse.

The equine hindgut microbiome is dominated by fibrolytic bacteria according to both culture-based^(^^41^^,^^42^^)^ and culture-independent studies^(^^43^^,^^44^^)^. Fibrolytic bacteria are represented in both the Firmicutes and Bacteroidetes phyla^(^^45^^)^. *Fibrobacter succinogenes*, *Ruminococcus flavefaciens* and *R. albus* are the most extensively studied fibrolytic bacteria in herbivores^(^^43^^,^^44,^^46^^)^ and, of these, *F. succinogenes* and *R. flavefaciens* represent 12 and 4 %, respectively, of total hindgut bacteria in the horse^(^^43^^,^^44^^,^^47^^)^. Due to their role in breaking down the most abundant carbohydrate in the forage-based equine diet, these bacterial species may play a causative role in the condition of equine obesity or overweight. Despite the interest in equine obesity^(^^8^^,^^9^^,^^48^^,^^49^^)^ and reliance on gut microbes for energy harvest, no studies to date have compared the abundance of Firmicutes, Bacteroidetes or fibrolytic bacteria in overweight *v.* moderate-condition mares.

A relationship between gut microbes or microbial products with obesity would be significant as hindgut microbes can provide more than 50 % of daily digestible energy (DE) requirements to a horse^(^^16^^,^^27,^^50^^)^, as compared with only 10% of the energy requirements of humans^(^^51^^–^^55^^)^. Alterations in the gut microbiota or changes in function of the gut microbes, such as enhanced VFA production, may influence body weight or adiposity in the horse despite similar energy consumption. In the present study, we assessed the *in vivo* diet digestibility of grass hay in overweight and moderate-condition mares. In addition, faecal and plasma VFA concentrations were measured to evaluate primary metabolic outputs of hindgut microbial fibre fermentation. Finally, abundance of members of the Firmicutes and Bacteroidetes phyla and the abundance of the fibrolytic bacteria *R. flavefaciens* and *F. succinogenes* in the faeces were measured. We evaluated the ratio of active, fibrolytic^(^^56^^)^ *R. flavefaciens* and *F. succinogenes* (16S ribosomal RNA (rRNA)) *v.* the total number of fibrolytic bacterial copies (16S ribosomal DNA (rDNA)) abundance, providing a measurement of the proportion of actively replicating bacteria. We hypothesised that overweight mares would have higher apparent hay digestibility and higher faecal and plasma acetate concentrations than moderate-condition mares. We also hypothesised that overweight mares will have an increased abundance of faecal Firmicutes and a lower abundance of Bacteroidetes. Furthermore, we expected overweight mares to have a higher abundance of active *R. flavefaciens* and *F. succinogenes* compared with moderate-condition mares.

## Materials and methods

### Animals and housing

A total of five moderate-condition adult, non-pregnant mares (body condition score (BCS) 5–6 on a nine-point scale^(^^57^^)^; age 7–20 years; weight 523–611 kg) and five overweight adult, non-pregnant mares (BCS 7–9/9; age 7–20 years; weight 511–575 kg) from the Virginia-Maryland Regional College of Veterinary Medicine teaching herd were allocated to the study. The total herd of twenty-two horses had been managed for at least 3 years, grouped by BCS and managed on the same pastures (rotated around cool-season grass pastures) and fed the same cool-season grass hay during winter months. Forage was fed at a rate to meet National Research Council^(^^58^^)^ daily DE requirements for ideal body weight. The overweight mares maintained a BCS > 6/9 throughout the year and the moderate-condition mares maintained a BCS < 6/9 throughout the year. Average BCS at the start of the study for the overweight and moderate-condition groups were 7·3/9 and 5·3/9, respectively. The study was designed to detect a 0·03 difference ± 0·015 sd^(^^59^^)^ in digestibility based on α of 0·05 and 1 – β of 0·885.

Mares were housed in individual box stalls (3·6 m × 3·6 m) with adjacent individual dry paddocks (3·6 m × 4·8 m) during the 15 d study. The study was divided into two periods: a 10 d acclimatisation period followed by a 4 d digestibility trial and a final day for morphometric measurements. For the first 10 d of the study, stalls were bedded with pine shavings and mares had access to paddocks 24 h per d. Stalls were cleaned twice daily (11.00 and 20.00 hours). For the last 5 d of the study, stalls were bare except for rubber mats; mares had access to outside paddocks 10–15 min once daily during the 11.00-hour thorough stall cleaning; faeces were accounted for as described below.

All horses received routine veterinary care including vaccines, deworming and dental floating before the study. The study was conducted during June 2011 in Blacksburg, VA (mean ambient temperature 22·1°C). The animals were maintained and all procedures were performed in accordance with the Virginia Tech Institutional Animal Care and Use Committee (IACUC) guidelines (IACUC no. 10-152-CVM).

### Diet

Before the study, all mares were housed on and allowed *ad libitum* access to the same cool-season grass (predominantly tall fescue; *Festuca arundinacea*) pastures all year round. Mares were fed cool-season grass hay (predominantly orchard grass (OG; *Dactylis glomerata*) hay) in the winter months when pasture forage was unavailable. Mares were housed in individual stalls during the study (days 0–15) and limit-fed a commercially available OG hay (Standlee Hay Company) (Table 1). Hay was fed in hay nets at 20 g/kg body weight as fed per d divided into two equal feedings at 08.00 and 20.00 hours. The mares were acclimatised to the hay during a 10 d acclimatisation period, as previously described^(^^60^^)^. Mares were offered a vitamin–mineral supplement (EquiMin^®^ Granular; Southern States) free choice, as previously fed on pasture; the supplement was withdrawn on days 11–15. Orts were weighed, recorded and subtracted from the daily amount of hay offered. Total daily DM intake (DMI) was determined for each mare by multiplying total hay intake by average hay DM.

**Table 1.** Nutrient analysis* of the orchard grass (*Dactylis glomerata*) hay fed to mares during the study

Analyte	Orchard grass hay	Equi-analytical reference interval†
DM (g/100 g as fed)	92·4	90·6–93·3
Digestible energy		
MJ/kg DM	8·46	7·54–9·21
Mcal/kg DM‡	2·02	1·8–2·2
CP (g/100 g DM)	14·5	6·9–14·7
ADF (g/100 g DM)	41·7	34·4–43·6
NDF (g/100 g DM)	62·8	56·5–69·9
WSC (g/100 g DM)	5·8	6·5–15·2
ESC (g/100 g DM)	2·9	4·6–10·3
Starch (g/100 g DM)	0·4	0·9–3·7
NSC (g/100 g DM)§	6·2	8·0–17·7
Ca (g/100 g DM)	0·46	0·3–0·7
P (g/100 g DM)	0·45	0·2–0·3

CP, crude protein; ADF, acid-detergent fibre; NDF, neutral-detergent fibre; WSC, water-soluble carbohydrates (monosaccharides, dissacharides, fructan oligo/polysaccharides); ESC, ethanol-soluble carbohydrates (monosaccharides, disaccharides); NSC, non-structural carbohydrates.

* Forage analysis performed was as previously described^(^^33^^)^; values include a single measurement on a composite sample.

† Equi-Analytical Laboratories; grass hay analyte reference range is mean ± 1 sd for 10000–40000 samples based on analyte.

‡ Digestible energy (kcal/kg DM) calculated as 2118 + 12·18 (CP %) – 9·37 (ADF %) – 3·83 (hemicellulose %) + 47·18 (fat %) + 20·35 (NSC) – 26·3 (ash %)^(^^93^^)^.

§ NSC (calculated) = WSC + starch.

### Sample collection

On day 0 and day 15, mares were body condition scored, weighed on a digital scale (Cambridge Scale Works) and assessed for subcutaneous fat (rump fat) thickness before the 08.00 hours feeding. BCS^(^^57^^)^ was subjectively scored on day 0 and day 15 by a single individual (M. L. S.). Rump fat thickness was measured with a 12 mHz tendon probe with the probe placed in the sagittal plane 5 cm off of midline at the centre of the pelvis^(^^61^^)^; measurements were taken in triplicate and averaged. All measurements were taken before the morning meal; day 0 measurements were taken immediately after transport to the research barn.

A 20 g hand grab sample of the commercially available OG hay was obtained twice daily at each feeding on days 11–14 and stored in individual brown paper bags until analysis. Total daily faeces were collected continuously throughout the day on days 11–14 into plastic bags, kept closed between collection, to prevent moisture loss of faeces; total collections were weighed four times daily (14.00, 20.00, 02.00 and 08.00 hours) before disposal. Additional three times daily (08.00–09.00, 14.00–15.00, and 20.00–21.00 hours) 200 g fresh faecal samples, for digestibility and gross energy analysis, were collected into tin mini loaf pans (Schneider Paper Products, Inc.) and placed in a plastic bag until processing within 2 h of collection. In addition, three 50 g faecal samples were collected once daily (08.00 hours) for VFA and microbial abundance analysis. Two samples were stored in empty 50 ml tubes (VWR International); one sample was stored in a 50 ml tube (VWR International) containing 25 ml RNAlater^®^ (Life Technologies) as per the manufacturer's protocol. The 50 g faecal sample and 25 ml RNAlater^®^ were manually homogenised immediately after collection. The 50 g faecal samples were placed immediately on ice, and stored at –80°C within 1 h of collection until further analysis. All 08.00 hours samples were collected from the rectum; the 14.00 and 20.00 hours samples were collected from the floor immediately after defecation (seconds after defecation was observed).

The 20 g OG hay samples and 200 g faecal samples were weighed and dried in a 55°C forced-air oven (Precision Freas Mechanical Convection Ovens Model 645; Pacific Combustion) for 96 h to achieve <10 g/100 g moisture. Dried OG hay and faecal samples were ground using a 1-mm screen (model 4 Wiley Mill; Thomas Scientific), composited within horse, and subsampled within horse. All digestibility analyses were evaluated in duplicate. Faecal output was calculated as the summed weight of the four daily total faecal collections and 200 g three times daily faecal samples for each horse.

Before faecal collection and feeding at 08.00 hours, blood samples were drawn into 10 ml tubes (BD Vacutainer^®^) containing lithium heparin for VFA analysis. Plasma was harvested within 30 min of collection after centrifugation (3000 ***g***) and stored at –80°C until analysis. Plasma samples were pooled within horse over the four sampling days and analysed for acetate in duplicate.

### Apparent digestibility

*In vivo* apparent diet DE digestibility and DM digestibility are used to represent total-tract digestibility while neutral-detergent fibre (NDF) apparent digestibility and acid-detergent fibre (ADF) apparent digestibility represent microbial fermentation in the hindgut. Gross energy of ground OG hay and faeces was measured with a bomb calorimeter (Parr 1271A Auto Calorimeter) using a sample size of 0·15–0·20 g (analysis was corrected for sample weight) and jacket temperature at 30°C; 1 g benzoic acid was used as the standard and 0·45–0·50 g mineral oil was used as the spike. Commercially available OG hay DE for each horse was calculated using the following:

DE (kJ/kg DM (kcal/kg DM)) = (gross energy of OG hay (kJ/kg DM (kcal/kg DM)) × total daily hay consumption (kg DM)) – (gross energy faeces (kJ/kg DM (kcal/kg DM)) × total daily faecal production (kg DM)).

Data are reported as kJ/kg DM (kcal/kg DM). DM, ash, ADF and NDF, inclusive of ash, were determined using AOAC procedures^(^^62^^)^. Apparent digestibility of DM was calculated with the following: DM digestibility = (DMI – faecal output)/DMI^(^^63^^)^; calculations were repeated for organic matter, NDF and ADF fractions.

### Volatile fatty acids

Frozen 50 g faecal samples were thawed at 4°C for 4 h and prepared as described by Otto *et al.*^(^^64^^)^. Briefly, 2 g of thawed faeces were mixed with 8 ml deionised water and 0·5 ml concentrated HCl (Fisher-Scientific), vortexed for 10 s, and then centrifuged at 25314 ***g*** for 20 min. The supernatant fraction was filtered through a 0·22 µm filter (Millipore Co.) and stored in 3·7 ml (1 fluid dram; DR) glass vials (no. 0333922B; Fisher Scientific). Samples were pooled to combine by day within horse and stored at –80°C until VFA analysis. Thawed pooled plasma and faecal supernatant fraction samples were spiked with 100 µl internal standard/volume marker (2·5 mm-[1,2-^13^C_2_]sodium acetate, 1 mm-[1,2,3-^13^C_3_]propionic acid, 1 mm-[1,2,3,4-^13^C_4_]sodium butyrate) then derivatised using a water, acetonitrile and 2-chloroethanol solution adapted from Kristensen^(^^65^^)^. Faecal preparations were analysed for acetate, propionate and butyrate, and plasma was analysed for acetate by GC and MS^(^^65^^)^.

### Faecal bacterial abundance

Frozen 50 g faecal samples were thawed at 4°C for 4 h before DNA extraction. A commercial kit (ZR Soil Microbe DNA MicroPrep™; Zymo Research) was used to extract DNA from 0·25 g homogenised and pelleted faeces as described by Shepherd *et al.*^(^^30^^)^.

Two storage methods (RNAlater^®^-preserved faeces and liquid N_2_-preserved faeces) and extraction kits (RNeasy^®^ Mini Kit, Qiagen, Ca and Zymo Soil/Faecal RNA Mini Prep, Zymo Research, Irving, CA) with and without a DNase step were evaluated with the goal of optimising RNA yield and quality from faeces. RNA quality was determined using a Bioanalyser 2100 (Agilent Technologies, Inc.). The highest 23S:16S ratio of 1·5 and RNA integrity number (RIN) of 8·4, indicators of RNA quality, were obtained from RNA extracted using the RNeasy^®^ Kit (Qiagen) with bead beating and DNase treatment. Furthermore, this method produced the cleanest 23S and 16S peaks and minimal noise (no additional peaks) on electropherograms. Therefore, RNA was extracted from 0·25 g of each faecal sample stored in RNAlater^®^ at –80°C after having thawed on ice for 2 h and strained to remove RNAlater^®^. The RNeasy^®^ Mini Kit Fungal/Plant protocol with on-column DNase was followed according to the manufacturer's instructions (Qiagen).

Extracted DNA and RNA concentrations were assessed by spectrophotometry (NanoDrop ND-1000 Spectrophotometer; Coleman Technologies). DNA and RNA was re-extracted from a faecal sample only when concentrations were <10 ng/µl. DNA was standardised to a concentration of 60–70 ng/µl; RNA was standardised to a concentration of 65–75 ng/µl.

Quantitative real-time PCR was used to quantify the abundance of total bacteria and members of the Firmicutes or Bacteroidetes phylum using previously designed primers (Table 2). Each 25 µl reaction contained 12·5 µl HotStart-IT^®^ SYBR^®^ Green qPCR Master Mix 2X (no. 75770; USB Corp.) with 5 mm-MgCl_2_, 0·4 mm-nucleotides and 10 nm-fluorescein in addition to 1·3 µl each of 16S rDNA forward and reverse primers (10 µm; Table 2), 2·5 µl 10 % dimethyl sulfoxide (Fisher-Scientific), additional 1 µl 25 mg/ml MgCl_2_, 0·5 µl ROX^TM^ passive reference dye (no. 75768; USB Corp.) and 4·9 µl nanopure nuclease-free water (no. E476; Amresco Inc.). The PCR protocol consisted of denaturation at 95°C for 3 min followed by forty cycles of 95°C for 15 s, annealing for 30 s (see annealing temperature in Table 2), and elongation at 72°C for 30 s. The melt curve consisted of 95°C for 1 min, 55°C for 1 min, seventy-one cycles of 60·5°C for 30 s increasing the temperature with each repeat. The melt curve was evaluated for a single fluorescent peak per PCR reaction; multiple fluorescent peaks indicate non-specific primer amplification (i.e. primer dimer formation).

**Table 2.** Primers used for determining bacterial abundance

Primer/probe and reference	Sequence (5′ → 3′)	Melting temperature (°C)	Target group and standard
926 F^(^^94^^)^	AAA CTC AAA KGA ATT GAC GG	49·9	Universal (*Exiguobacterium* spp.)
1062 R^(^^94^^)^	CTC ACR RCA CGA GCT GAC	55·9	
Firm928 F^(^^94^^)^	TGA AAC TYA AAG GAA TTG ACG	49·9	Firmicutes (*Exiguobacterium* spp.)
Firm1040 R^(^^94^^)^	ACC ATG CAC CAC CTG TC	55·2	
Cfb798 F^(^^94^^)^	CRA ACA GGA TTA GAT ACC CT	49·9	Bacteroidetes (*Flavobacterium* spp.)
Cfb967 R^(^^94^^)^	GGT AAG GTT CCT CGC GTA T	54·1	
Universal F^(^^95^^)^	TCCTACGGGAGGCAGCAGT	60·0	Universal
Universal – probe^(^^95^^)^	CGTATTACCGCGGCTGCTGGCAC	60·0	
Universal F^(^^95^^)^	GGACTACCAGGGTATCTAATCCTGTT	60·0	
*Ruminococcus flavefaciens* F^(^^44^^)^	GTGTCGTGAGATGTTGGGTTAAGT	60·0	*R. flavefaciens*
*R. flavefaciens* – probe^(^^44^^)^	CCGCAAAGAGCGCAACCCTT	60·0	
*R. flavefaciens* R^(^^44^^)^	AGTGCTCTTGCGTAGCAACTAAAG	60·0	
*Fibrobacter succinogenes* F^(^^44^^)^	CGTTCCCGGGCCTTGT	60·0	*F. succinogenes*
*F. succinogenes* – probe^(^^44^^)^	CACACCGCCCGTCAAGCCATG	60·0	
*F. succinogenes* R^(^^44^^)^	CACGACTTAGAGCACTCCCTTCTC	60·0	

F, forward; R, reverse.

Abundance of *R. flavefaciens* and *F. succinogenes* was determined using TaqMan^®^ primers and probes (Table 2) as previously designed by Hastie *et al.*^(^^44^^)^. Each 20 µl reaction contained 65 ng DNA, 10 µl HotStart-IT Probe qPCR Master Mix 2x (no. 75770; USB Corp.), 1 µl of 20X TaqMan^®^ assay (AB TaqMan^®^ Assay; Table 2), 0·4 µl of ROX^TM^ as passive reference dye (no. 75768; USB Corp.) and 7·6 µl nanopure nuclease-free water (no. E476; Ameresco Inc.). PCR conditions consisted of one cycle of 2 min at 95°C for activation of HotStart-IT polymerase, followed by thirty-five cycles of denaturation at 95°C for 15 s, primer annealing and real-time detection at 60°C for 30 s, and extension at 72°C for 1 min carried out with a 7300 real-time PCR detection system (Applied Biosystems). Standard curves were constructed using 6-fold dilutions of target DNA from pure cultures of *R. flavefaciens* S85 and *F. succinogenes* FD-1 provided by Dr Roderick Mackie (University of Illinois, Urbana). Absolute abundance was calculated as log_10_ copies/g faeces. The abundance of active *F. succinogenes* and *R. flavefaciens* was determined as described above except that 70 ng RNA were used as the starting material and converted to complementary DNA by the addition of 0·2 µl Moloney murine leukemia virus (M-MLV) RT (no. 75783; USB Corp.) and 0·2 µl RNase inhibitor (no. 75782; USB Corp.). PCR conditions as described above were preceded by one cycle of 5 min at 50°C for reverse transcription of RNA before amplification. Negative controls, without complementary DNA and reverse transcriptase, were run to rule out DNA contamination. Each reaction was prepared and carried out in biological and technical duplicates as described^(^^66^^)^ using an ABI 7300 (Applied Biosystems).

### Statistical analysis

Duplicate digestibility, plasma and faecal VFA, and bacterial abundance analyses were conducted on samples pooled within horse for the collection period. Data were analysed using SAS (version 9.2; SAS Institute Inc.). A GLIMMIX procedure was used for analysis, with mare within group as the subject, using the following model for analysis: 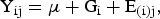 where Y_ij_ = dependent variables DMD, NDFD, ADFD, plasma acetate, faecal acetate, propionate and butyrate, and abundance of total bacteria, Firmicutes, Bacteroidetes, *F. succinogenes* and *R. flavefaciens*; µ = the mean of Y; G_i_ = fixed effect of group (overweight and moderate condition); and E_(i)j_ = random effect of mare within group. For each model, residual plots were inspected to verify the assumption that errors followed a normal distribution with a constant variance. Differences between groups were considered significant with *P* < 0·05. Data are presented as mean values with their standard errors.

## Results

### Animals and apparent digestibility

Body weight, BCS, rump fat thickness and DMI for the two groups are presented in Table 3. Body weight (*P* = 0·35) did not differ between groups; however, BCS was higher (*P* < 0·01) and mean rump fat was larger (*P* = 0·03) in overweight mares. DMI did not differ between groups (*P* = 0·61). DM, NDF and ADF apparent digestibilities did not differ between groups (Table 4).

**Table 3.** Mare body weight (BW), body condition score (BCS) and rump fat thickness measured on days 0 and 15 and DM intake (DMI) during days 11–14 (Mean values with their standard errors)

	Overweight mares (*n* 5)		Moderate-condition mares (*n* 5)		Group
	Mean	sem	Mean	sem	*P*
BW (kg)	538	30	561	43	0·352
BCS*	7·3	0·3	5·3	0·3	<0·001
Rump fat (cm)	2·3	0·3	1·6	0·4	0·026
DMI (g/kg BW)	17·5	1·1	17·1	1·6	0·610

* BCS measured on a scale of 1 to 9.

**Table 4.** Hay digestibility in overweight and moderate-condition mares during days 11–14 of the study (Mean values with their standard errors)

	Overweight mares (*n* 5)		Moderate-condition mares (*n* 5)		Group
	Mean	sem	Mean	sem	*P*
Digestible energy					0·37
MJ/kg DM	9·42	0·59	9·13	0·34	
Mcal/kg DM	2·25	0·14	2·18	0·08	
Digestibility (g/100 g)					
DM	0·56	0·03	0·54	0·02	0·191
OM	0·58	0·00	0·56	0·02	0·189
NDF	0·60	0·03	0·59	0·03	0·506
ADF	0·55	0·03	0·54	0·03	0·514

OM, organic matter; NDF, neutral-detergent fibre; ADF, acid-detergent fibre.

### Volatile fatty acids

Faecal acetate, propionate and butyrate concentrations did not differ significantly between overweight and moderate-condition mares (Table 5). However, mean plasma acetate concentration was higher (*P* = 0·034) in the overweight mares (1·55 (sem 0·10) mmol/l) than the moderate-condition mares (1·39 (sem 0·11) mmol/l).

**Table 5.** Volatile fatty acid concentrations in the faeces (mg/g dry faeces) and plasma (mmol/l) of overweight and moderate-condition mares on days 11–14 of the study (Mean values with their standard errors)

	Overweight mares (*n* 5)		Moderate-condition mares (*n* 5)		Group
	Mean	sem	Mean	sem	*P*
Faeces					
Acetate	8·28	1·76	8·31	1·40	0·973
Propionate	4·44	1·35	4·70	1·33	0·770
Butyrate	0·68	0·17	0·68	0·19	0·972
Plasma					
Acetate	1·55	0·10	1·39	0·11	0·034

### Faecal bacterial abundance

The abundance of total bacterial 16S rRNA copies, as determined using TaqMan^®^ primers/probes, was higher (*P* < 0·001) than with SYBR^®^ Green primers (Table 6). There was no statistically significant difference in the abundance of total bacteria, Firmicutes and Bacteroidetes 16S rDNA, as determined using SYBR^®^ Green primers, from the faeces of overweight *v.* moderate-condition mares (Table 6). A difference in the average Firmicutes:Bacteroidetes ratio in the overweight (2·76 (sem 0·46)) and moderate-condition (3·09 (sem 0·35)) mares was not detected (*P* = 0·588). Differences in total bacteria, *R. flavefaciens* or *F. succinogenes* 16S rDNA and 16S rRNA abundance, as determined using TaqMan^®^ primers/probes, were not detected between overweight *v.* moderate-condition mares (Table 6). The 16S rRNA:16S rDNA ratio was higher (*P* < 0·001) for *F. succinogenes* than for all bacteria and *R. flavafaciens* in both groups (Table 6).

**Table 6.** Abundance (log_10_ copies 16S ribosomal DNA (rDNA) and 16S ribosomal RNA (rRNA)/g faeces) and 16S rDNA:16S rRNA ratios for total bacteria, Firmicutes, Bacteroidetes, *Ruminococcus flavefaciens* and *Fibrobacter succinogenes* (Mean values with their standard errors)

	Overweight mares		Moderate-condition mares		Group
	Mean	sem	Mean	sem	*P*
Log_10_ copies 16S rDNA/g faeces					
Total bacteria†	9·07	0·08	9·14	0·06	0·505
Total bacteria‡	8·38	0·03	8·38	0·02	0·824
Firmicutes†	8·89	0·08	9·07	0·08	0·125
Bacteroidetes†	8·48	0·07	8·60	0·04	0·180
*R. flavefaciens*‡	6·77	0·09	6·82	0·05	0·643
*F. succinogenes*‡	5·32	0·11	5·54	0·06	0·142
Log_10_ copies 16S rRNA /g faeces‡					
Total bacteria	6·88	0·06	7·05	0·09	0·152
*R. flavefaciens*	5·13	0·05	5·35	0·12	0·131
*F. succinogenes*	4·96	0·20	4·97	0·16	0·956
16S rRNA:16S rDNA ratio‡					
Total bacteria	0·82	0·01	0·84	0·01	0·158
*R. flavefaciens*	0·76	0·01	0·78	0·01	0·156
*F. succinogenes*	0·93*	0·04	0·90*	0·02	0·421

* Mean value of 16S rRNA:16S rDNA ratio was significantly different from that for *R. flavefaciens* (*P* < 0·001).

† Bacterial abundance was determined by the use of SYBR^®^ Green primers (see Table 2).

‡ Bacterial abundance was determined by the use of TaqMan^®^ primers/probes (see Table 2).

## Discussion

### Animals and apparent digestibility

The mares in the present study were chosen because they had been under the same management and feeding practices for the past 3 or more years before the study yet displayed variable body-condition phenotypes. No differences in DM or fibre digestibilities were detected between overweight and moderate-condition mares in the present study despite all fractions being numerically higher in the overweight mares. A difference in the mean hay DE between overweight and moderate-condition mares of 251 kJ/kg hay DM (0·06 Mcal/kg hay DM) translates to an additional 1025 MJ DE (245 Mcal DE)/year in a 500 kg overweight mare fed 20 g/kg BW per d hay, DM basis or 10 kg DM per d. The additional energy could conceivably be stored as fat. Generally speaking, a 32·2 MJ (7·70 Mcal) energy surplus/deficit is needed to gain/lose 1 kg body weight^(^^67^^)^. This estimate does not take into account differences between fat and lean tissue gained/lost during weight gain/loss; however, the 1026 MJ DE (245 Mcal DE)/year could hypothetically lead to an increase of 32 kg body weight in 1 year's time.

Ragnarsson & Jansson^(^^59^^)^ reported a 0·03 difference in haylage digestibility between six Standardbred and six Icelandic horses (0·57 *v.* 0·54). Increased individual variation in digestibility was anticipated in the present study, as compared with the Ragnarsson study, due to breed variation. Furthermore, the facilities in the present study allowed for a maximum of ten horses to be evaluated during the same period to avoid the effect of time/period. We used a 10 d adaptation period and 4 d collection period as this is a standard approach in equine digestibility trials^(^^60^^)^. We do not anticipate that provision of a longer adaptation period would have allowed us to detect a difference in digestibility. However, based on the variation in the present study, we would need a larger cohort of obese (*n* 14) and lean (*n* 14) adult mares to detect a 0·02 difference in grass hay DM digestibility.

The overweight mares may, if allowed *ad libitum* access, consume more total daily DM than the moderate-condition mares, thereby influencing total daily energy intake. We did not evaluate voluntary OG hay consumption in the overweight *v.* moderate-condition mares and thus are unable to comment on the effect of voluntary intake on the overweight condition. Conversely, we cannot comment on the potential effect of voluntary overconsumption on digestibility.

### Volatile fatty acids

Faecal VFA concentrations in the present study (Table 5) were higher than concentrations in the faeces of geldings limit-fed lucerne cubes (2·84 mg acetate, 0·89 mg propionate and 0·55 mg butyrate/g faecal DM) as reported by Hussein *et al.*^(^^17^^)^. This difference could be due to an effect of diet (lucerne *v.* hay) or individual variation in VFA production by hindgut microbes or VFA absorption. Argenzio *et al.*^(^^15^^)^ reported that total VFA concentrations varied from 20 to 60 mmol/l in the hindgut among ponies fed the same pelleted feed. Other VFA, such as valerate, isovalerate and isobutyrate, were not evaluated in the present study as they collectively represent less than 10 % of total VFA in horse faeces^(^^17^^)^. Therefore, a comparison of the VFA ratios in the present study and prior studies cannot be made.

Plasma acetate concentrations in the present study were higher (>1·0 mmol/l; Table 5) than previously reported in horses (0·56 (sem 0·07) mmol/l)^(^^68^^)^ and human subjects (<0·1 mmol/l)^(^^69^^)^. The adult Standardbreds in the Waller *et al.* study^(^^68^^)^ were managed on a sweet feed and forage diet *v.* forage-only diet in the present study. The exact cause and significance of higher plasma acetate in the overweight *v.* moderate-condition mares were beyond the scope of the study. Possible causes for increased plasma acetate in overweight mares could be due to increased microbial VFA production, reduced microbial acetate utilisation/metabolism^(^^70^^,^^71^^)^, increased VFA absorption across the gut mucosa, reduced acetate oxidation, increased hepatic acetate production^(^^72^^,^^73^^)^ or reduced hepatic TAG synthesis. VFA absorption is negatively correlated with gut lumen pH and positively associated with the concentration of a given VFA in the lumen^(^^12^^)^. Diet influences hindgut and faecal lumen pH, with non-structural carbohydrates (mono/disaccharides, starches, fructans), as found in grains, favouring a more acidic pH^(^^18^^,^^41,^^74^^,^^75^^)^. Reductions in hindgut pH may lead to enhanced VFA absorption secondarily due to mucosal barrier compromise, which could lead to the horse's demise^(^^76^^,^^77^^)^. Mares in the present study were fed a grass-hay diet low in non-structural carbohydrates^(^^33^^)^ with no inclusion of grains. Cani & Delzenne^(^^78^^)^ described a cascading process of obesity, altered gut microbes, altered gut barrier function, metabolic endotoxaemia and subsequent inflammation in human models. We did not evaluate the entire microbial population in the present study; however, we did not find evidence of altered gut microbes between overweight and moderate-condition mares.

The potential effects of increased plasma acetate in overweight horses are numerous. Of the VFA, peripheral tissues directly utilise acetate as an energy source^(^^27^^)^. Once in the blood, acetate may be aerobically oxidised and directly used for energy^(^^27^^)^ or stored as TAG in adipose and skeletal tissue^(^^28^^)^. Once absorbed, acetate can be oxidised via the tricarboxylic acid (TCA) cycle or stored in the form of adipose, as acetate is the primary substrate for *de novo* fat synthesis in the horse^(^^28^^)^. Furthermore, acetate may directly increase hepatic lipogenesis, lipoprotein lipase and subsequent fat storage as found in the murine model^(^^78^^)^. In humans, acetate is used as a substrate for *de novo* fatty acid synthesis in adipose tissue^(^^79^^)^ and VFA are recognised as a link between obesity and gut microbes in human subjects^(^^80^^)^. Therefore, the cause and significance of higher plasma acetate in overweight *v.* moderate-condition mares should be further explored. VFA may secondarily influence energy intake by influencing food intake. In ruminants, rumen infusions of acetate decrease intake^(^^81^^,^^82^^)^. Similarly, rectal acetate infusion increased plasma peptide YY, which generally inhibits food intake, in human subjects^(^^69^^)^. To our knowledge the effects of parenteral or rectal acetate infusions have not been evaluated in horses; we feel that this would be worth investigating. Furthermore, as previously discussed, we did not evaluate voluntary intake in the present study and thus cannot evaluate a potential relationship between plasma acetate and intake.

Faecal VFA concentrations do not accurately reflect those in the caecum and colon^(^^15^^)^. Therefore, without a direct measurement of caecal/colon VFA concentrations, we cannot speculate whether a difference in caecal/colon VFA concentrations would be detected in this cohort. Similarly, plasma VFA concentrations in the jugular vein will not accurately represent microbial VFA production or even portal vein VFA concentrations, particularly for propionate and butyrate. Most absorbed propionate is converted into glucose by the liver and provides 50–61 % of blood glucose in horses^(^^83^^)^, and butyrate is the preferred energy substrate for colonocytes and thus plays an important role in the maintenance of hindgut health. Therefore, of the venous VFA, we focused on plasma acetate. Measurement of portal venous VFA concentration in conjunction with caecal and colon VFA concentrations would be a more accurate method to elucidate VFA absorption rates *in vivo* and thus determine if a difference in VFA absorption exists between overweight and moderate-condition horses. Portal vein catheterisation in horses has been described^(^^84^^)^ and can be placed in conjunction with caecal cannulas; however, both are invasive procedures.

### Faecal bacterial abundance

The relative abundance of Firmicutes in the hindgut is positively associated with obesity in human subjects^(^^21^^,^^36^^)^, pigs^(^^34^^)^ and rodents^(^^20^^,^^35^^)^. However, a difference in abundance of Firmicutes was not detected between overweight and moderate-condition adult mares in the present study (Table 6). Furthermore, the faecal Firmicutes:Bacteroidetes ratio in the faeces of overweight *v.* moderate-condition mares did not differ. We hypothesise that *R. favafaciens* and *F. succinogenes* 16S rRNA abundance would be higher in the overweight *v.* moderate-condition mares as an indication of higher fibrolytic bacterial activity. However, we did not detect a difference in the abundance of *R. favafaciens* and *F. succinogenes* in overweight *v.* moderate-condition mares.

The higher 16S rRNA:16S rDNA ratio for *F. succinogenes* may represent a higher activity of *F. succinogenes* in the rectum of adult mares fed grass hay as compared with *R. flavafaciens*. *F. succinogenes* plays an important fibrolytic role in herbivores fed a grass-based diet^(^^85^^)^. *F. succinogenes* has superior fibrolytic activity as compared with *Ruminococcus* spp.^(^^86^^,^^87^^)^, which may explain the increased 16S rRNA:16S rDNA ratio, a representation of activity. Furthermore, time of day, with respect to feeding, influences the abundance of *F. succinogenes* in the rumen of dairy cattle^(^^88^^)^. However, the effect of time or feeding was not evaluated in the present study, as faecal samples for microbial analysis were collected once daily, before the morning feeding.

The higher abundance of total bacteria as determined using SYBR^®^ Green primers *v.* TaqMan^®^ primers/probes (Table 6) is probably due to the higher specificity when using TaqMan^®^ primer/probe combinations^(^^89^^)^. TaqMan^®^ probes bind only between the two PCR primers; therefore, the complimentary sequence must be present for the primers to bind and subsequently the probe to bind and result in a fluorescent signal and thus TaqMan^®^ probes are indicated when evaluating bacterial members in low abundance within a population^(^^90^^)^. In contrast, SYBR^®^ Green binds and fluoresces with any double-stranded DNA; SYBR^®^ Green primers are less expensive than TaqMan^®^ probes, can be used with a wider range of primers, and are commonly used when evaluating bacterial members in high abundance within a population^(^^90^^)^.

This is the first study, to the authors' knowledge, of evaluating 16S rDNA abundance alongside 16S rRNA abundance to characterise the equine faecal bacterial population with an emphasis on fibrolytic bacteria. Evaluation of 16S rDNA abundance has been used to evaluate the abundance of bacteria in equine gut/faecal samples^(^^44^^)^, but does not distinguish between viable and non-viable bacteria. The 16S rRNA abundance and can be used as an indicator of bacterial activity^(^^91^^)^ and is typically higher than 16S rDNA abundance in pure culture^(^^91^^,^^92^^)^. As reported in the present study, we expected the 16S rRNA abundance to be lower than the 16S rDNA abundance in faeces because RNA is a less stable molecule than DNA. Furthermore, both viable and non-viable bacteria are present in faeces of adult mares and the latter would not be transcribing the rDNA into rRNA. We used faecal samples as a non-invasive means to evaluate two different groups of horses; however, we cannot directly infer that the findings presented here represent the caecal and colonic microbiome.

In conclusion, overweight mares have higher plasma acetate concentrations than lean mares fed the same commercially available OG hay diet. The cause and significance of this finding should be further explored as a mechanism linking obesity and gut microbes.
